# Exploring MRI and Mammography Lesion Features for Breast Cancer Detection in PTEN Hamartoma Tumor Syndrome

**DOI:** 10.3390/cancers17050856

**Published:** 2025-03-02

**Authors:** Alma Hoxhaj, Annemieke Milants, Porjai Techanithisawat, Peter Bult, Nicoline Hoogerbrugge, Ritse M. Mann

**Affiliations:** 1 Department of Medical Imaging, Radboud University Medical Center, 6500 HB Nijmegen, The Netherlands; ritse.mann@radboudumc.nl; 2 Department of Radiology, The Netherlands Cancer Institute, Antoni van Leeuwenhoek Hospital, 1066 CX Amsterdam, The Netherlands; 3 Radboud Institute for Health Sciences, Radboud University Medical Center, 6525 GA Nijmegen, The Netherlands; 4 Sint-Niklaas Vitaz, 9100 Sint-Niklaas, Belgium; 5 Queen Sirikit Centre for Breast Cancer, Bangkok 10330, Thailand; 6 Department of Pathology, Radboud University Medical Center, 6525 GA Nijmegen, The Netherlands; 7 Department of Human Genetics, Radboud University Medical Center, 6525 GA Nijmegen, The Netherlands; nicoline.hoogerbrugge@radboudumc.nl; 8 European Reference Network Genetic Tumour Risk Syndromes (ERN GENTURIS), 6525 GA Nijmegen, The Netherlands

**Keywords:** *PTEN*, PHTS, cancer, breast cancer surveillance, magnetic resonance imaging, mammography

## Abstract

(1) Background/Objectives: Women with *PTEN* hamartoma tumor syndrome (PHTS) face a significantly increased risk of breast cancer (up to 66%) and a high prevalence of benign breast lesions (30–75%), which can complicate cancer detection and underscore the need for effective surveillance strategies. This study aimed to evaluate the imaging characteristics of breast cancers and benign breast lesions using magnetic resonance imaging (MRI) and mammography, with the goal of improving early cancer detection, reducing unnecessary biopsies, and guiding future surveillance protocols. (2) Methods: This retrospective single-institution study included 65 PHTS women aged ≥18 years (2001–2021), 39 of whom participated in a high-risk breast cancer surveillance program. Imaging features of breast cancers from MRI and mammography (when available) and of benign breast lesions from MRI only were assessed independently by two breast radiologists and correlated with pathology reports. Sensitivity and performance of MRI and mammography in detecting breast cancers and benign breast lesions were analyzed using descriptive statistics and correlation analyses, with significance set at *p* < 0.05. (3) Results: Imaging was available for re-evaluation for 17 breast cancers (with MRI available for 10 cases and mammography for 15 cases) diagnosed in 11 women and 31 benign breast lesions (with MRI available for 29 cases and mammography for 26 cases) in 16 women. MRI identified 90% (9/10) of the breast cancers for which it was available as suspicious, with malignant features retrospectively identifiable in 50% of baseline scans. In comparison, mammography identified only 40% (6/15) of breast cancers and was notably less effective in women with dense breast tissue. For benign breast lesions, MRI identified all lesions (29/29), while mammography underperformed, correctly identifying only 58% (15/26). However, ambiguous enhancement features on MRI occasionally posed challenges in distinguishing between benign breast lesions and malignancies. (4) Conclusions: MRI significantly outperformed mammography in accurately characterizing both breast cancers and benign breast lesions in women with PHTS, particularly in younger women with dense breast tissue. These findings reinforce the critical role of MRI as the primary surveillance tool for this high-risk population, given that breast cancers in women with PHTS tend to exhibit typical malignant features on MRI. However, they also highlight the importance of careful interpretation of MRI findings for benign breast lesions and the need for additional strategies to minimize unnecessary interventions.

## Simple Summary

Women with *PTEN* hamartoma tumor syndrome (PHTS) face a high risk of developing breast cancer, yet the specific imaging characteristics of breast cancer and benign breast lesions in this population are poorly understood. This study provides an initial perspective on these imaging features using magnetic resonance imaging (MRI) and mammography to improve early detection and reduce unnecessary biopsies. The findings show that MRI is highly effective for characterizing breast cancer in women with PHTS, while mammography performs less well, particularly in younger women with dense breast tissue. However, benign breast lesions in women with PHTS partially exhibit features associated with malignancies on MRI imaging, complicating diagnosis and leading to higher biopsy rates. These results underscore the importance of MRI as the primary surveillance tool for women with PHTS and suggest that mammography may not need to be introduced until later in life. However, they also emphasize the importance of careful interpretation of MRI findings for benign breast lesions and the need for additional strategies and further research to improve diagnostic accuracy and minimize unnecessary interventions.

## 1. Introduction

*PTEN* hamartoma tumor syndrome (PHTS) encompasses a spectrum of clinical manifestations resulting from pathogenic germline mutations in the tumor suppressor gene *PTEN* [1,2,3]. Women with PHTS face distinct health challenges, including a markedly high cumulative lifetime risk of breast cancer, which can reach up to 66% [4]. Among this population, breast cancer is the most common hereditary cancer [5,6,7]. Consequently, women with PHTS adhere to more stringent surveillance guidelines than the general population [8,9]. The American Cancer Society (ACS) [10] and European expert opinion guidelines [11] recommend initiating annual breast magnetic resonance imaging (MRI) surveillance at age 25, with the addition of supplemental mammography starting at age 30 [12].

Recent studies have demonstrated the effectiveness of breast cancer surveillance in women with PHTS. Notably, the cancer detection rate (CDR) achieved through annual surveillance is impressive (45 per 1000 rounds (95% CI 20–94)). Both the sensitivity (100%) and specificity (82%) of the combined modalities were found to be high [13]. Consistent with findings from studies in high-risk populations (MRI: 71–96%, mammography: 19–51%) [14,15,16], MRI demonstrated superior sensitivity (100%) compared to mammography (50%) when these modalities were assessed independently [13]. Similarly, despite the individual specificity of mammography (92%) and MRI (84%) being adequate, MRI alone was responsible for the diagnosis of breast cancer in three out of five women who underwent both imaging modalities. Although this is a preliminary observation based on a limited number of cases and requires further validation, the findings align with those observed in women with BRCA1/2 pathogenic variants [17,18], suggesting that the added value of mammography in these high-risk groups might be limited.

Significant research has focused on breast cancer imaging features in breast MRI and mammography in women with BRCA1/2 pathogenic variants [19,20,21,22], as well as in the general population [23,24,25]. However, the specific imaging characteristics of breast cancers in women with PHTS remain underexplored. Given the relatively high prevalence of benign breast lesions (46%) in this population [13], studies that detail both benign and malignant imaging features could help improve the accuracy of surveillance programs by enhancing sensitivity and specificity. Furthermore, such data could provide valuable insights for refining expert-opinion-based guidelines and optimizing imaging strategies, particularly for young women with PHTS.

The primary objective of this study was to characterize the imaging features and corresponding pathological outcomes of breast disease in women with PHTS. By identifying the most common imaging characteristics of breast disease in this high-risk population, the study aims to enhance radiologists’ ability to promptly detect breast cancer and distinguish them from benign breast lesions. This could help reduce recall and biopsy rates and, most importantly, support the early detection of breast cancer.

## 2. Materials and Methods

Radboud University Medical Center, a recognized national and European expert center for PHTS and a member of the European Reference Network on Genetic Tumour Risk Syndromes (ERN GENTURIS), identified women aged 18 or older with either a confirmed pathogenic or likely pathogenic *PTEN* variant (N = 62) or a variant of uncertain significance accompanied by a clear PHTS phenotype (N = 3) between January 2001 and February 2021 for this retrospective single-institution study [26].

**Figure 1:**
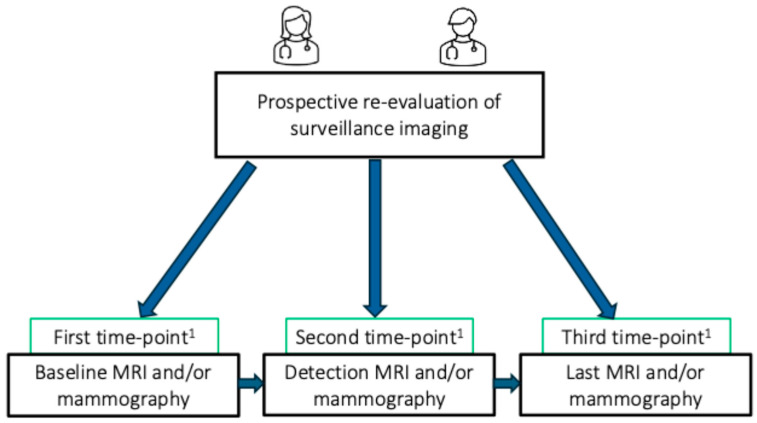
Flowchart illustrating the prospective re-evaluation of imaging examinations for women enrolled in the surveillance program. ^1^ These timelines occasionally overlapped. The detection time point coincided with the baseline examination for one woman, when the woman was diagnosed with breast cancer during her initial surveillance visit.

Women with PHTS who started surveillance at our institution (N = 39) were monitored within our high-risk breast cancer surveillance program, in line with the ACS guidelines [10] and the national PHTS guidelines [27]. From October 2020 to December 2021, two specialized breast radiologists, with 4 and 3 years of experience, respectively, prospectively re-evaluated surveillance imaging at three key time points, when available (Figure 1): baseline, detection, and final surveillance scans. Imaging examinations were evaluated using the BI-RADS lexicon (5th edition) [28].

When available, surveillance mammograms were reviewed alongside MRIs to compare lesion visibility and/or assess the presence of additional findings. For women not enrolled in the surveillance program (*N* = 26), detection mammograms, when available, were re-evaluated. When a discrepancy in categorical values was noted, a majority vote based on the original report was used to resolve this. For continuous values, if the discrepancy was <5 mm, the mean value between assessments of the two readers was used, while if the discrepancy was >5 mm, the mean between the assessment of the original report and the assessment of the reader closer to the original report was calculated.

Except for the knowledge of a malignancy being present in the cancer detection imaging examination, the radiologists were blinded to any other clinical information. MRI and mammography images were reviewed concurrently. For benign breast lesions, imaging features were assessed exclusively on MRI, while mammography (when available) was used solely to evaluate lesion detectability. Due to the prevalence of multiple benign breast lesions in women with PHTS (often >20 per side), only the three most significant lesions (e.g., largest, most distinctive) were described for each case. MRI protocols, while varying over time, consistently included T1 weighted pre- and post-contrast exams, adhering to the European Society of Breast Imaging standards [29].

Assessments on a dedicated breast MRI workstation (Dynacad, Invivo, USA) included scoring lesion detection, size (measured along the longest axis to allow for direct comparison with the longest axis reported in pathology reports), morphology, and enhancement kinetics. Morphologic assessments covered lesion shape, margin appearance, and enhancement patterns. Lesion enhancement kinetics were evaluated according to the criteria described by Kuhl et al. [30]. Mammography assessment, using a full-field digital machine, included two views (mediolateral–oblique and craniocaudal). Breast composition, lesion detection, lesion size (measured along the longest axis, consistent with MRI for cases where MRI was not available), morphology, and associated features were evaluated and scored.

Corresponding pathology reports were retrieved to assess the nature of imaging findings, with histopathological data sourced from local or national archives. Parameters included tumor size and type. In cases where the tumor was a carcinoma of no special type (NST), grade and receptor statuses (ER, PR, HER2) were retrieved. At our institution, these parameters were identified exclusively for NST tumors. For benign breast lesions, histological types were noted.

Statistical analysis employed descriptive statistics for patient and tumor characteristics. The kappa statistic, intraclass correlation coefficient (ICC), or Bland–Altman plot was used to assess inter-observer reliability based on the type of data. The strength of agreement beyond chance for different κ/ICC values was rated as: poor (<0.40), fair (0.40–0.59), good (0.60–0.74), excellent (0.75–1.00). Bootstrapping was used to calculate 95% confidence intervals (CIs) for kappa values using 1000 replications. Significance was set at a two-sided *p*-value below 0.05, using R software version 1.2.5001 for analyses [31].

## 3. Results

**Figure 2:**
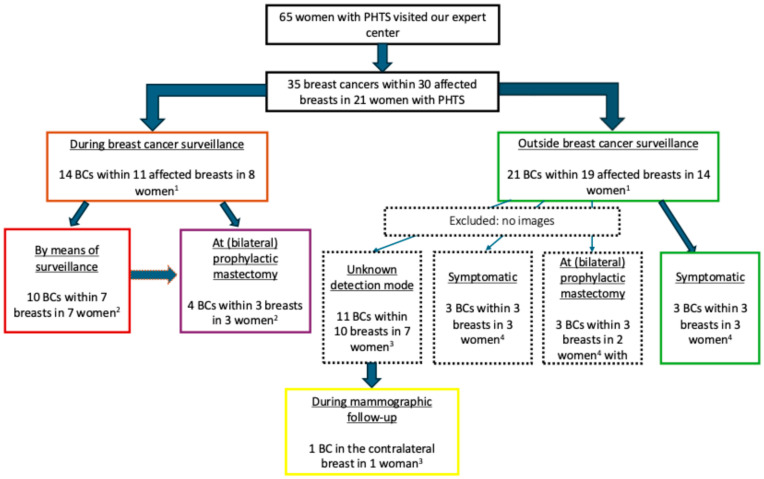
Flowchart of the distribution of breast cancers with imaging examinations available for re-evaluation. ^1^ One woman was diagnosed with breast cancer before undergoing genetic testing, which revealed that she was a *PTEN* variant carrier. After this diagnosis, she enrolled in the surveillance program. ^2^ Two women were diagnosed with 2 BCs in 2 breasts by means of surveillance and opted for contralateral prophylactic mastectomy which revealed a contralateral BC. ^3^ One woman previously diagnosed with one BC (unknown detection mode) was later diagnosed with a contralateral BC during mammographic follow-up. ^4^ One woman opted for contralateral prophylactic mastectomy because of prior diagnosis of a symptomatic BC.

In total, 35 breast cancers were diagnosed in 21 (32%) out of the 65 women identified with PHTS (median age at first breast cancer diagnosis, 40 years (range, 24–59 years)). Imaging examinations were available for re-evaluation for 17 (49%) breast cancers diagnosed in 11 women (52%) (Figure 2).

Of these 17 breast cancers, pathological analysis identified 10 as invasive carcinomas of NST, 5 as ductal carcinomas in situ (DCIS), 1 as an invasive lobular carcinoma (ILC), and 1 as an invasive papillary carcinoma.

Among the 17 breast cancers with imaging examinations available for re-evaluation, 3 breast cancers were diagnosed in three symptomatic women outside the surveillance program prior to their identification as pathogenic *PTEN* variant carriers. For these women, only mammography examinations were available for re-evaluation.

**Figure 3:**
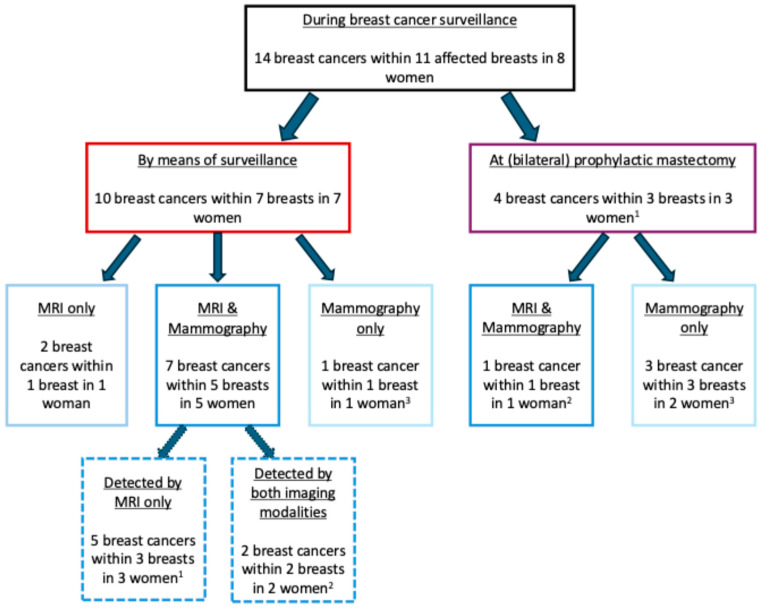
Flowchart of breast cancers detected by means of surveillance. ^1^ One woman was diagnosed with two biopsy-proven malignant lesions, both detected via MRI and not visible on mammography. A third breast cancer in the same breast was subsequently identified during mastectomy. ^2^ One woman was diagnosed with one breast cancer through imaging examinations, visible on both MRI and mammography. Following her decision to undergo prophylactic mastectomy of the contralateral breast, an incidental BC was discovered in the contralateral breast. ^3^ One woman was diagnosed with one BC identified through imaging, with only mammography performed. After opting for prophylactic mastectomy of the contralateral breast, an incidental BC was also detected in this breast.

The remaining 14 breast cancers were diagnosed in eight women enrolled in the surveillance program. Ten of these fourteen breast cancers were detected in seven women through surveillance. In detail, two breast cancers in one patient were identified solely through MRI (mammography was performed elsewhere and unavailable for re-evaluation), while one breast cancer in another patient was diagnosed using only mammography (MRI was contraindicated due to a neurostimulator). Furthermore, seven breast cancers were diagnosed in five women who had both MRI and mammography detection examinations. Among these ten breast cancers detected in women undergoing surveillance, one breast cancer was diagnosed in the prevalent round (baseline examinations) in one woman and nine breast cancers during incident rounds (i.e., diagnosed at subsequent surveillance rounds) in six women. The median interval time between baseline and detection imaging examinations was 2 years (range, 0–5 years), with no interval cancers observed (Figure 3).

Lastly, four breast cancers were incidentally identified at pathology in three women enrolled in the surveillance program who opted to undergo (bilateral) prophylactic mastectomy. Of these, one breast cancer was identified in a woman who had undergone both MRI and mammography; the remaining three breast cancers were identified in two women who had only mammography examinations available. These breast cancers were not detected during surveillance nor described at imaging re-evaluation.

To summarize, regardless of whether women were enrolled in the surveillance program at the time of breast cancer diagnosis, MRI examinations were available for re-evaluation in 10 cases, while mammography was available for 15 cases.

### 3.1. Inter-Observer Reliability

Breast density was classified as B (36%), C (46%), and D (18%). The kappa statistic indicated poor agreement for breast density assessment (κ = 0.37 (95% CI −0.08–0.82)). In contrast, the kappa statistic indicated good inter-observer reliability for BI-RADS score assessment (κ = 0.67 (95% CI 0.19–1.0)).

Twelve breast cancers were described during the re-evaluation of imaging examinations by both readers, and one breast cancer by only one reader. The remaining four breast cancers, not identified by either radiologist during the re-evaluation, were the incidental breast cancers detected at pathology following (bilateral) prophylactic mastectomy. In light of this, the kappa statistic revealed excellent agreement in assessing lesion visibility (κ = 0.85 (95% CI 0.0–1.0)) and lesion type (κ = 0.86 (95% CI 0.32–1.0)).

**Figure 4:**
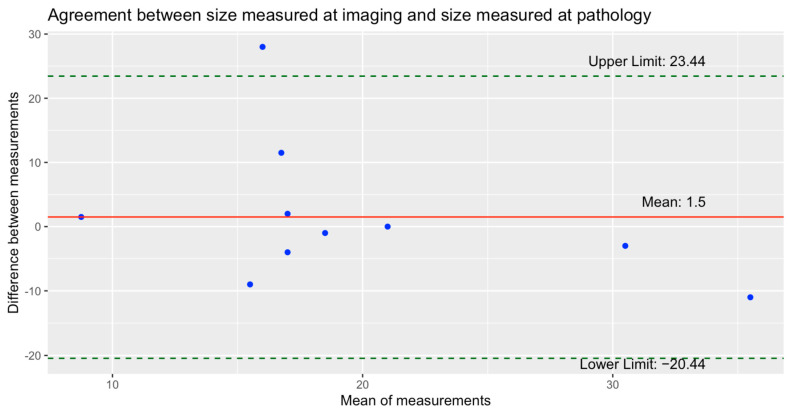
Bland-Altman plot for the agreement between sizes measured at imaging and sizes measured at pathology.

Pathological size data were available for 11 of these 12 breast cancers described by both readers, as one patient was treated with neo-adjuvant chemotherapy and achieved a pathological complete response (pCR). One case was excluded due to inconsistent size estimations between imaging and pathology. This breast cancer was detected through mammography, when mammography was the only modality available, and was characterized as an area of global asymmetry measuring 118 mm. However, pathology revealed an invasive carcinoma NST measuring only 30 mm. The median pathological size of these 10 breast cancers included in the analysis was 19 mm (range, 2–41), compared to a median imaging size of 19.5 mm (range, 9.5–30), with size differences ranging from −11 to +28 mm. The inter-observer reliability for sizes measured at imaging was excellent (ICC = 0.99 (0.97–1.0)). The agreement between sizes measured at imaging and at pathology is shown in the Bland–Altman plot (Figure 4).

### 3.2. Magnetic Resonance Imaging Features and Corresponding Pathology Outcome of Breast Cancers

MRI examinations of 10 breast cancers diagnosed in six women enrolled in the breast cancer surveillance program (median age at detection MRI, 45 years (range, 31–55 years)) were available for re-evaluation. Of these, one breast cancer was incidentally discovered during a contralateral prophylactic mastectomy in a woman previously diagnosed with breast cancer. During the re-evaluation of the images, this incidental breast cancer was not described either.

**Figure 5:**
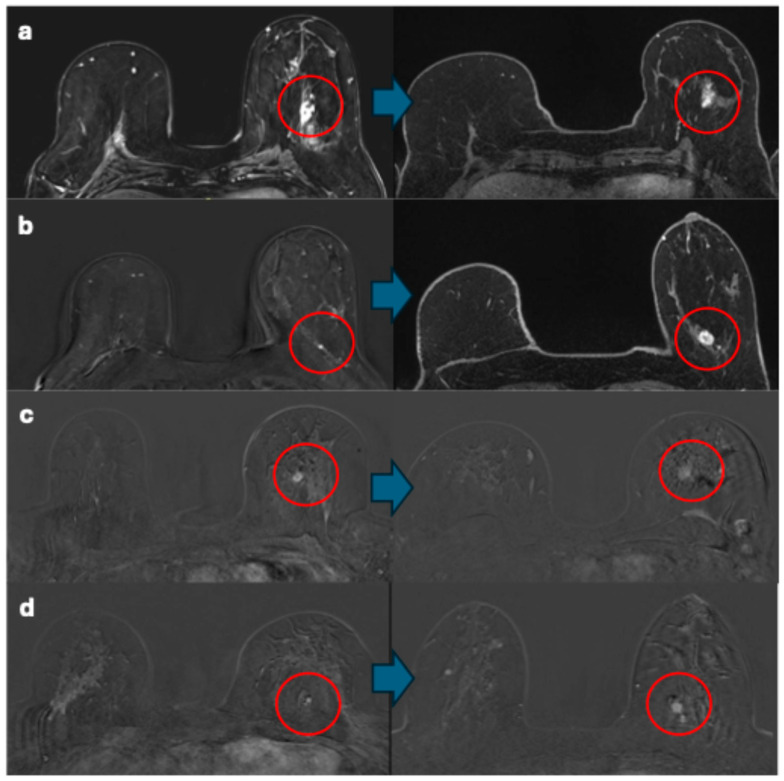
(**a**) One breast cancer showed no change in size (30 mm), features (non-mass enhancement), or enhancement curves (type III) from baseline to detection. (**b**) One breast cancer showed changes in size, features, and enhancement curves from baseline (5 mm, focus, type I) to detection (18 mm, oval mass, type III). (**c**,**d**) Two breast cancers exhibited changes in features and enhancement curves from baseline (oval masses, type I) to detection (irregular masses, type III). Red circles indicate the breast cancers. Blue arrows connect the baseline images to the detection images.

The remaining nine breast cancers were identified through surveillance (one in the prevalent round and eight in incident rounds) and described during the re-evaluation of the images. Of the eight breast cancers detected in incident rounds, four breast cancers were identified by both radiologists in both the baseline and detection MRI examinations. The median time between baseline and detection examinations for these breast cancers visible at baseline was 1.5 years (range, 1–2 years). In the comparison of baseline to detection MRIs, the lesions were visible but not necessarily deemed suspicious (Figure 5).

Table 1 presents the MRI features and pathological outcomes of the nine breast cancers described during the re-evaluation of detection examinations. Among these breast cancers, six were carcinomas of NST, one was a DCIS, one was an ILC, and one was an invasive papillary carcinoma. Of the six carcinomas of NST, three were triple-negative breast cancers (50%) while tumor grades were evenly distributed between intermediate (50%) and high (50%) grades.

### 3.3. Mammographic Imaging Features and Corresponding Pathology Outcome of Breast Cancers

Mammography examinations for 15 breast cancers diagnosed in 10 women (median age at detection mammography, 40.5 years (range, 30–55 years)) were available for re-evaluation. Of these 15 breast cancers, 4 were incidentally discovered during prophylactic mastectomy and were not described during the re-evaluation of the images either.

Among the remaining 11 breast cancers described during the mammography re-evaluation, 3 breast cancers were diagnosed in three symptomatic women outside the surveillance. Eight breast cancers were detected through surveillance (one breast cancer in the prevalent round and seven in incident rounds); of these, one breast cancer had only mammography examinations available, while the remaining seven had both MRI and mammography examinations. However, five out of these seven breast cancers in women with both imaging modalities were only visible on MRI, consistent in both the original reports and the re-evaluation.

**Figure 6:**
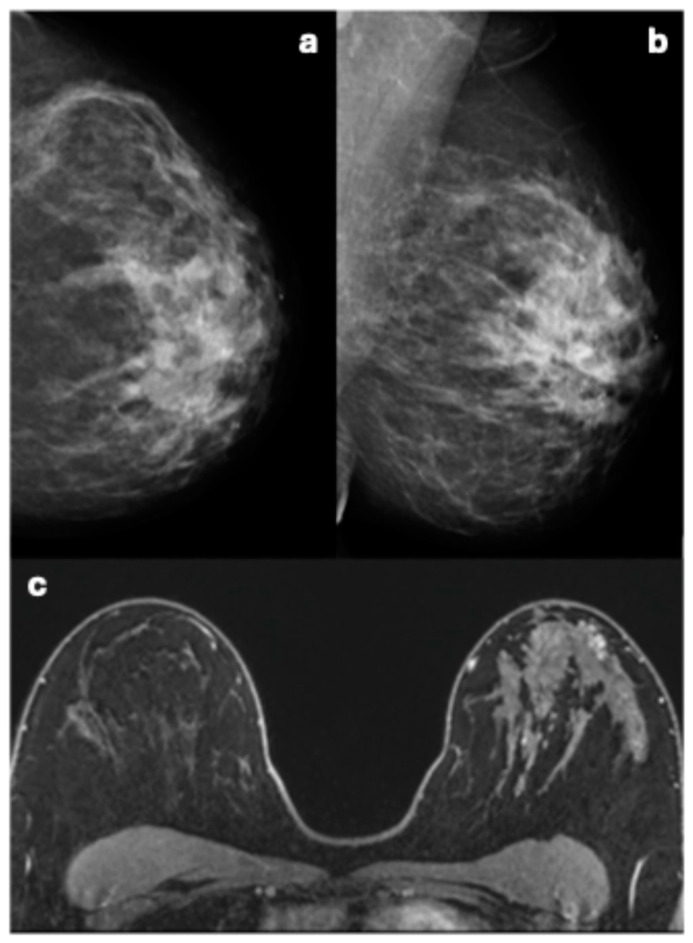
Imaging of a woman with dense breasts illustrating the limitations of mammography and the sensitivity of MRI. (**a**) Cranio-caudal and (**b**) medio-lateral oblique mammography views show no identifiable or suspicious lesion. However, MRI (**c**) reveals a segmental, clumped non-mass enhancement (NME) lesion in the left breast, characterized by a type III kinetic curve. Pathology subsequently confirmed this lesion to be ductal carcinoma in situ (DCIS).

These five breast cancers undetected by mammography occurred in three women with breast density classified as C (n = 1) or D (n = 2). These breast cancers were described at MRI as irregular masses (N = 3) with irregular margins and heterogeneous enhancement patterns (three invasive carcinomas NST), one focal non-mass enhancement (NME) with heterogeneous internal enhancement (invasive carcinoma NST), and one segmental NME with clumped internal enhancement (DCIS) (Figure 6).

In four of the five breast cancers not visible at mammography, non-suspicious round/oval masses, resembling fibroadenomas, were described at the re-evaluation of MRI examinations.

Table 2 presents the mammographic features and pathological outcomes of the six breast cancers identified during the re-evaluation of detection mammography examinations. Among these breast cancers, four were carcinomas of NST, one was a DCIS, and one was an invasive papillary carcinoma. Of the four carcinomas of NST, three were triple-negative breast cancers (75%) while tumor grades were either intermediate (25%) or high (75%).

### 3.4. Imaging Features and Corresponding Pathology Outcome of Benign Breast Lesions

**Figure 7:**
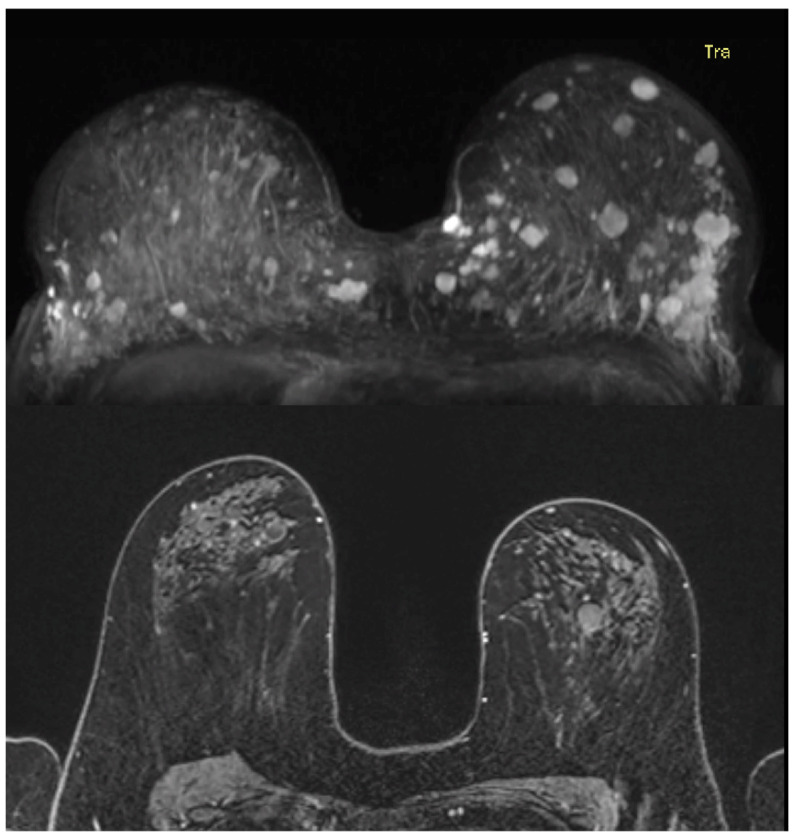
MRI examinations of two women with multiple benign breast lesions in both breasts.

In total, 89 distinct pathologically confirmed benign breast lesions were diagnosed within 23 women (35%), with a median age of 38 years at first diagnosis (range, 15–61 years). Imaging examinations for 31 benign breast lesions in 16 women were available for re-evaluation. In five of these cases, multiple (>20 per side) well-defined benign breast lesions were radiologically identified during the re-evaluation of the imaging examinations (Figure 7). For these women, only the three most significant lesions were described following the BI-RADS lexicon.

MRI examinations for 29 benign breast lesions detected in 14 women were available for re-evaluation. The MRI imaging features and the types of lesions are listed in Table 3. Of these, corresponding mammography examinations were available for 26 benign breast lesions. However, 11 benign breast lesions were not detectable in mammography and were only identified in MRI. In total, 15 (57%) of the benign breast lesions, found in nine women, were also visible in mammography. Additionally, three benign breast lesions were described in women for whom only mammography examinations were available.

## 4. Discussion

This study characterizes the imaging and pathological features of breast cancer and benign breast lesions in women with PHTS, a high-risk population that remains underexplored in breast imaging literature. Our findings highlight the critical role of MRI in breast cancer surveillance, while underscoring the diagnostic challenges posed by overlapping imaging features of breast cancers and benign breast lesions in this population.

All seven breast cancers with both MRI and mammography imaging examinations available for re-evaluation were identified on MRI both at the time of the original imaging examination and during the re-evaluation, confirming the high sensitivity of breast MRI in detecting breast cancers in women with PHTS. This is consistent with its established superiority in high-risk populations [32,33]. In contrast, only two out of the seven breast cancers with both imaging modalities were identified on mammography at both the time of the original imaging examination and during the re-evaluation. Mammography’s limited sensitivity can be attributed, in part, to the high prevalence of dense breast tissue in this cohort, which is known to obscure cancers [34]. Notably, 64% of the women diagnosed with breast cancer had breast density category C or D [35].

In our study, readers demonstrated poor agreement in breast density assessment (κ = 0.37) but good agreement in BI-RADS score assessment (κ = 0.67). These findings align with prior studies showing substantial variability among radiologists in using final assessment categories [36,37], highlighting the need for specific training programs to increase inter-observer reliability [38]. However, agreement was excellent for both lesion visibility (κ = 0.85) and lesion type (κ = 0.86), as was the inter-observer reliability for measuring lesion sizes through imaging (ICC = 0.99). Differences between sizes measured at imaging and pathology (Figure 4) were shown to be relatively small, with imaging overestimating the lesion size by an average of 1.5 mm. Most data points clustered around the mean difference line and remained within the limits of agreement, further demonstrating the consistency of imaging-based measurements. Pathological size data were available for 10 breast cancers, with lesion size measured at MRI in 9 cases and at mammography in only 1 case. This distribution reinforces the idea that MRI may be superior to mammography for accurate size estimation [39], as the high inter-observer reliability for MRI measurements and the minimal differences between imaging and pathological sizes support its precision. These findings reinforce the role of breast MRI as the preferred imaging modality for size estimation in high-risk populations, such as women with PHTS.

Most breast cancers identified on MRI appeared as masses (56%) with irregular shapes (60%), irregular margins (80%), and heterogeneous enhancement (80%). NMEs were also common (44%), with heterogeneous internal enhancement patterns in the majority (75%). All breast cancers with available kinetic curve data exhibited type III (washout) patterns, which are typical of malignancy. The MRI imaging features of breast cancers in women with PHTS were largely consistent with those seen in young women at average risk [23,24,25] but differed from breast cancers in BRCA1/2 carriers [19] and particularly from those associated with BRCA1 variants. Unlike BRCA1-associated breast cancers, which often present as rounded masses with sharp margins and rim enhancement on MRI [20], PHTS-associated breast cancers more frequently exhibited irregular shapes and margins, as well as type III (washout) enhancement curves, characteristics known to be associated with malignancy [30].

The majority of these breast cancers presented at mammography as masses (67%) characterized by irregular shapes (75%), spiculated margins (50%), and high density (100%). Architectural distortion (56%) was the most frequently associated mammographic feature. The absence of suspicious calcifications in breast cancers among women with PHTS, together with the high prevalence of dense breast tissue, likely further explains the reduced detection rates observed with mammography in our study. This finding aligns with results in BRCA1 carriers, where cancers similarly often lack suspicious calcifications [22,40]. These findings highlight the critical role of MRI as the primary imaging modality for effective breast cancer surveillance in this high-risk population.

Pathologically, most breast cancers detected in our cohort were invasive cancers (85%), with only 15% being DCIS. Among the invasive cancers, 54% exhibited high nuclear grade, and 37% were triple negative (TN). These rates are intermediate between those reported in BRCA1 carriers (57% high grade, 85% TN) and BRCA2 carriers (56% high grade, 23% TN) [41], suggesting differences in tumor biology between PHTS and BRCA-associated breast cancers. The intermediate incidence of TN breast cancers, which are less frequently associated with DCIS [42,43,44] and, in turn, with microcalcifications on mammography in approximately 75–90% of cases [45,46], further supports the notion that the added value of mammography in PHTS surveillance may be limited. These findings might support increasing the starting age for mammography in this population, relying primarily on MRI for early breast cancer detection.

Benign breast lesions in this cohort predominantly appeared on MRI as round or oval masses (68%) with circumscribed margins (80%) and exhibited either heterogeneous enhancement (60%) or dark internal septations (36%). Most benign breast lesions demonstrated type I or type II kinetic curves, with the most common histopathological diagnoses being fibroadenomas (45%) and fibrocystic changes (18%), aligning with existing MRI descriptions of these conditions in the literature [47,48]. Notably, features typically associated with malignancy, such as heterogeneous enhancement [49], were frequently observed in benign breast lesions, complicating the differentiation of benign breast lesions and breast cancers based solely on imaging [13]. Therefore, in women with PHTS, the primary challenge posed by benign breast lesions might be the high rate of false positives. This highlights a limitation of breast MRI, which, despite its high sensitivity [50], can result in significant patient anxiety, unnecessary biopsies, and potential overtreatment due to false-positive findings. Encouragingly, studies have shown that increased radiologist expertise, the use of multiparametric breast MRI, and the integration of radiomics and machine and deep learning technologies for breast lesion classification can significantly reduce false positives and false negatives, thereby enhancing diagnostic accuracy and improving patient outcomes [51,52,53,54,55,56].

Our study has several limitations. The small sample size reflects the rarity of PHTS and limits the generalizability of our findings. Additionally, the single-institution, retrospective design introduces potential selection and reporting biases. Furthermore, as the study spanned 20 years, advancements in imaging technology and evolving protocols may have introduced variability in imaging quality and interpretation. Despite these limitations, our main objective was to describe the imaging features of breast cancers and benign breast lesions in this very specific high-risk yet understudied group of women. Importantly, BI-RADS criteria were consistently applicable across all scans, regardless of the time period. To enhance surveillance strategies for women with PHTS and reduce unnecessary recalls and biopsies, future research should prioritize data pooling from multiple centers to generate larger, more representative datasets.

## 5. Conclusions

This study provides initial insights into the imaging features of breast disease in women with PHTS, emphasizing the critical role of MRI in surveillance. Mammography’s limited performance in young women with dense breasts suggests its role should be carefully reconsidered, with a potential shift to initiating mammography at a later age. The overlapping imaging features of benign breast lesions and breast cancers highlight the diagnostic challenges and underscore the need for careful evaluation of MRI findings.

## Figures and Tables

**Table 1:** Features of Breast Cancers at Magnetic Resonance Imaging in Women with *PTEN* Hamartoma Tumor Syndrome.

Features		Breast Cancers (N = 9) Described at MRI Re-Evaluation
Background parenchymal enhancement		
	Minimal or mild	2/6
	Moderate or marked	4/6
Lesion type		
Mass	5	
Shape	Round	1/5
	Oval	1/5
	Irregular	3/5
Margins	Circumscribed	1/5
	Irregular	4/5
Enhancement	Heterogeneous	4/5
	Rim enhancement	1/5
Non-mass enhancement	4	
Distribution modifiers	Focal area	2/4
	Segmental	1/4
	Regional	1/4
Internal enhancement	Heterogeneous	2/4
	Clumped	2/4
Kinetic curve assessment: initial phase ^1^	Fast	7/7
Kinetic curve assessment: delayed phase ^1^	Washout	7/7
Type of breast cancer	NST	6/9
	ILC	1/9
	DCIS	1/9
	Other	1/9
ER ^2^	Positive	3/6
	Negative	3/6
PR ^2^	Positive	3/6
	Negative	3/6
HER2 ^2^	Positive	1/6
	Negative	5/6
Triple negative ^2^	No	3/6
	Yes	3/6
Grade ^2^	1	0/6
	2	3/6
	3	3/6

NST = invasive carcinoma of no special type; ILC = invasive lobular carcinoma; DCIS = ductal carcinoma in situ; ER = estrogen receptor; PR = progesterone receptor; HER2 = human epidermal growth factor receptor 2. ^1^ Kinetic curve assessment was available for 7 breast cancers with surveillance MRI examination available. ^2^ ER, PR, HER2 status, and grade were available for 6 NST breast cancers.

**Table 2:** Features of Breast Cancers at Mammography in Women with *PTEN* Hamartoma Tumor Syndrome.

Features		Breast Cancers (N = 6) Described at Mammography Re-Evaluation
Breast density	A	0
	B	3
	C	2
	D	1
Lesion type		
Mass	4	
Shape	Round	1/4
	Irregular	3/4
Margins	Circumscribed	1/4
	Indistinct	1/4
	Spiculated	2/4
Density	High	4/4
Asymmetry	1	
	Global	1/1
Calcifications	1	
Morphology	Fine pleiomorphic	1/1
Distribution	Regional	1/1
Associated features	9	
	Calcifications	1
	Nipple retraction	1
	Skin thickening	1
	Trabecular thickening	2
	Architectural distortion	4
Type of breast cancer	NST	4/6
	DCIS	1/6
	Other	1/6
ER ^1^	Positive	3/4
	Negative	1/4
PR ^1^	Positive	3/4
	Negative	1/4
HER2 ^1^	Positive	1/4
	Negative	3/4
Triple negative ^1^	No	3/4
	Yes	1/4
Grade ^1^	1	0/4
	2	1/4
	3	3/4

NST = invasive carcinoma of no special type; DCIS = ductal carcinoma in situ; ER = estrogen receptor; PR = progesterone receptor; HER2 = human epidermal growth factor receptor 2. ^1^ ER, PR, HER2 status, and grade were available for 4 NST breast cancers.

**Table 3:** Features of Benign Breast Lesions at Magnetic Resonance Imaging in Women with *PTEN* Hamartoma Tumor Syndrome.

Features		Benign Breast Lesions (N = 29) Described at MRI Examinations (N = 14)
Lesion type		
Focus	2	
Mass	25	
Shape	Round	9/25
	Oval	8/25
	Irregular	8/25
Margins	Circumscribed	20/25
	Irregular	5/25
Enhancement	Homogeneous	1/25
	Heterogeneous	15/25
	Dark internal septations	9/25
Non-mass enhancement	2	
Distribution modifiers	Focal area	1/2
	Regional	1/2
Internal enhancement	Heterogeneous	2/2
Kinetic curve assessment: initial phase ^1^	Slow	13/27
	Medium	8/27
	Fast	6/27
Kinetic curve assessment: delayed phase ^1^	Persistent	11/27
	Plateau	13/27
	Washout	3/27
Type of benign breast lesion	Fibroadenoma	13/29
	Fybrocystic changes	5/29
	Adenosis	4/29
	Fibrosis/sclerosis	3/29
	Radial scar	3/29
	Intraductal papilloma	1/29

^1^ Kinetic curve assessment was available for 1 focus, 24 mass lesions, and 2 non-mass enhancement.

## Data Availability

The data presented in this study are available on request from the corresponding author on reasonable request.
